# Expression of miR-27a-3p is an independent predictive factor for recurrence in clear cell renal cell carcinoma

**DOI:** 10.18632/oncotarget.4064

**Published:** 2015-05-27

**Authors:** Wataru Nakata, Motohide Uemura, Mototaka Sato, Kazutoshi Fujita, Kentaro Jingushi, Yuko Ueda, Kaori Kitae, Kazutake Tsujikawa, Norio Nonomura

**Affiliations:** ^1^ The Department of Urology, Osaka University Graduate School of Medicine, Osaka, Japan; ^2^ Laboratory of Molecular and Cellular Physiology, Osaka University Graduate School of Pharmaceutical Sciences, Osaka, Japan

**Keywords:** clear cell carcinoma, renal cell carcinoma, microRNA, prognosis, recurrence

## Abstract

MicroRNAs (miRNAs) are noncoding RNAs that regulate gene expression and function in tumor development and progression. We previously identified up-regulated miRNAs in clear cell renal cell carcinoma (ccRCC) compared to matched-pair normal kidney by microarray. Here, we identify miRNAs that are up-regulated in ccRCC and are also correlated with survival and/or recurrence. Twenty-four samples from ccRCC patients who underwent nephrectomies between 2011 and 2012 were divided into two groups: one of eleven patients who experienced recurrence (Group 1), and one of thirteen patients with no evidence of disease (Group 2) 2 years after surgery. Analyzing 22 miRNAs that were up-regulated in ccRCC in our previous study, we identify five miRNAs that were statistically up-regulated in Group 1 versus Group 2 by quantitative real-time PCR. We then evaluated these miRNAs in an independent cohort of 159 frozen ccRCC samples. High levels of miR-27a-3p (*p* < 0.01) correlated with a worse progression-free survival rate. Multivariate analysis revealed that miR-27a-3p was an independent predictive factor for recurrence. For functional analysis, miR-27a-3p controlled cell proliferation, migration and invasion in RCC cell lines. MiR-27a-3p could act as oncogenic miRNA and may be a candidate for targeted molecular therapy in ccRCC.

## INTRODUCTION

Renal cell carcinoma (RCC) accounts for 2–3% of all malignant tumors in adults, and clear cell renal cell carcinoma (ccRCC) is one of the most frequent RCC subtypes [1]. Early-stage ccRCC is curable by surgery [2], but approximately 6% of patients newly diagnosed as RCC present with metastases [3]. Although molecular-targeted drugs, such as the tyrosine kinase inhibitors (TKI) and inhibitors of the mammalian target of rapamycin (mTOR), are currently administered for the treatment of metastatic ccRCC, the effects of these drugs are limited and not curative [4]. The prognosis for metastatic ccRCC remains poor, with a 5-year survival rate < 10% [5]. In addition, around 30% of patients who undergo complete surgical resection of localized ccRCC eventually develop distant metastases [6]. The prognosis for recurrent ccRCC varies widely. Detecting early relapse can improve a patient's prognosis, because molecular-targeted drugs show the most effect when the metastatic burden is small, and surgical complete resection of single or limited numbers of metastasis can lead to longer survival [7]. Therefore, there is a great need to identify new prognostic biomarkers that, using surgical samples obtained during nephrectomy, can help predict recurrence in ccRCC.

MicroRNAs (miRNAs) are 20–24 nucleotide noncoding RNAs that negatively regulate gene expression by inhibiting translation of messenger RNA (mRNA) or targeting mRNAs for degradation. Currently, more than 1000 miRNAs have been identified in human cells (miRBase; http://www.mirbase.org/index.shtml), and these regulate an estimated 30% of all gene expression [8]. Aberrant miRNA expression is involved in tumorigenesis of several cancers, including ccRCC [9]. MiRNAs have been implicated to function as tumor suppressors or oncogenes. Previous studies have shown that decreased tumor suppressive miRNAs resulted in up-regulation of their target oncogenes, and increases in oncogenic miRNAs led to the reduction of their target tumor suppressor genes [10].

Several recent studies have used miRNA expression profiling to compare ccRCC to normal kidney [11, 12], to classify histological subtype of RCC [7], to identify early relapse after nephrectomy [13, 14], to detect circulating miRNAs in serum for early diagnosis [15], and to evaluate the potential association of miRNA-related SNPs with RCC risk and clinical outcome [16, 17]. Our group used microarray expression profiling to identify miRNAs that are up- or downregulated in ccRCC compared to matched normal kidney, as discussed in our previous study [18]. A few papers have focused on the association between miRNA expression and survival [19–21]. However, these studies used only a small number of patients, and the correlations between miRNA expressions and survival in ccRCC were not fully elucidated.

In the present study, we focused on up-regulated miRNAs in ccRCC compared to normal tissue, and evaluated the association of up-regulated miRNAs with cancer-specific survival rate and recurrence rate in ccRCC patients.

## RESULTS

### MiRNA expression in discovery cohort

We previously identified miRNAs up-regulated in ccRCC compared to matched-pair normal kidney using microarray analysis [18]. From this cohort, we selected 22 miRNAs that showed 2.5-fold higher expression in ccRCC than in normal tissue (Supplementary Table 1, Supplementary Figure 1).

Here, we evaluated the levels of these 22 candidate miRNAs using recent frozen samples for validation. There were 24 samples, stored in RNAlater, obtained from patients diagnosed with ccRCC at Osaka University Graduate School of Medicine between 2011 and 2012 (Table 1). We divided these into two groups, of which eleven patients were alive with disease or died of disease (AWD and DOD) and thirteen patients had no evidence of disease (NED) 2 years after surgery. qRT-PCR was performed on the samples to analyze the expression of the 22 candidate miRNAs. We compared the differences between the two groups using Wilcoxon test. Due to the small number of patients in our cohort, we considered miRNAs as potentially differentially expressed if *p* < 0.1. Under these parameters, miR-21a-3p, 193a-3p and 34a-5p were significantly up-regulated (*p* < 0.05), and miR-193b-5p and 27a-3p were statistically up-regulated (*p* < 0.1) in the DOD/AWD group compared to the NED group (Table 2). Thereafter, we evaluated these 5 miRNAs in further studies.

**Table 1:** Patient characteristics

		Discovery cohort	Validation cohort
**Number of patients**		24	159
**Age (years)**	**Median (range)**	63 (34–86)	65 (34–82)
**Gender**	**Male/female**	18/6	107/52
**TNM stage**	**I/II/III/IV**	10/0/5/9	104/18/18/19
**Grade**	**G1, 2/G3, 4**	19/5	139/20
**Vein invasion**	**Yes/no/unknown**	5/19/0	20/114/25
**Metastasis status**	**M0/M1**	15/9	140/19
**Prognosis**	**NED/AWD/DOD**	13/6/5	113/19/27
**Follow-up (month)**	**Median (IQR)**	17 (5–37)	60 (36–84)

NED: no evidence of disease, AWD: alive with disease, DOD: dead of disease, IQR: interquartile range

**Table 2:** MiRNAs differentially expressed in ccRCC in two groups (NED v.s AWD and DOD)

miRNA	NED Median (IQR)	AWD or DOD Median (IQR)	*P* value (Wilcoxon test)
**miR-21-3p**	0.91 (0.51–1.38)	1.88 (0.97–4.02)	0.010
**miR-193a-3p**	1.09 (0.62–1.46)	1.50 (1.27–2.42)	0.011
**miR-34a-5p**	1.15 (0.83–1.44)	1.90 (1.11–2.63)	0.014
**miR-193b-5p**	0.79 (0.67–1.17)	1.17 (0.78–2.85)	0.098
**miR-27a-3p**	1.37 (0.74–1.82)	1.95 (0.79–2.22)	0.098
**miR-224-3p**	1.06 (0.27–1.48)	1.62 (0.56–2.19)	0.13
**miR-21-5p**	0.50 (0.24–0.79)	2.16 (0.21–2.98)	0.16
**miR-15a-5p**	0.99 (0.71–1.11)	1.14 (0.84–1.70)	0.16
**miR-3128**	0.29 (0.19–0.42)	0.35 (0.26–1.10)	0.18
**miR-451a-3p**	0.91 (0.48–1.78)	0.56 (0.14–0.94)	0.20
**miR-34a-3p**	0.82 (0.49–1.41)	1.49 (0.49–3.32)	0.21
**miR-146b-5p**	0.58 (0.12–0.93)	1.08 (0.15–1.64)	0.25
**miR-106b-3p**	1.03 (0.85–1.54)	1.19 (1.01–2.28)	0.42
**miR-148a-3p**	0.73 (0.52–1.41)	1.38 (0.45–2.65)	0.42
**miR-92b-3p**	1.19 (0.68–2.37)	1.42 (1.06–2.43)	0.45
**miR-122-5p**	1.11 (0.67–1.66)	0.79 (0.27–1.97)	0.49
**miR-210-3p**	1.07 (0.74–1.71)	1.12 (0.84–1.84)	0.64
**miR-15b-5p**	1.11 (0.98–1.34)	1.14 (0.72–2.32)	0.77
**miR-1271-5p**	1.41 (0.59–2.09)	1.21 (0.66–2.68)	0.82
**miR-629-5p**	0.31 (0.24–0.69)	0.44 (0.18–0.74)	0.82
**miR-148b-3p**	0.81 (0.54–1.18)	1.03 (0.42–1.58)	0.86
**miR-155-5p**	N.D	N.D	-

NED: no evidence of disease, AWD: alive with disease, DOD: dead of disease, IQR: interquartile range, N.D: not detected

In confirmation of our microarray results, we found that these 5 miRNAs showed significantly higher expression by qRT-PCR in tumor samples compared to matched-pair normal kidney tissue (Supplementary Figure 2).

### MiRNA expression in validation cohort

**Figure 1:**
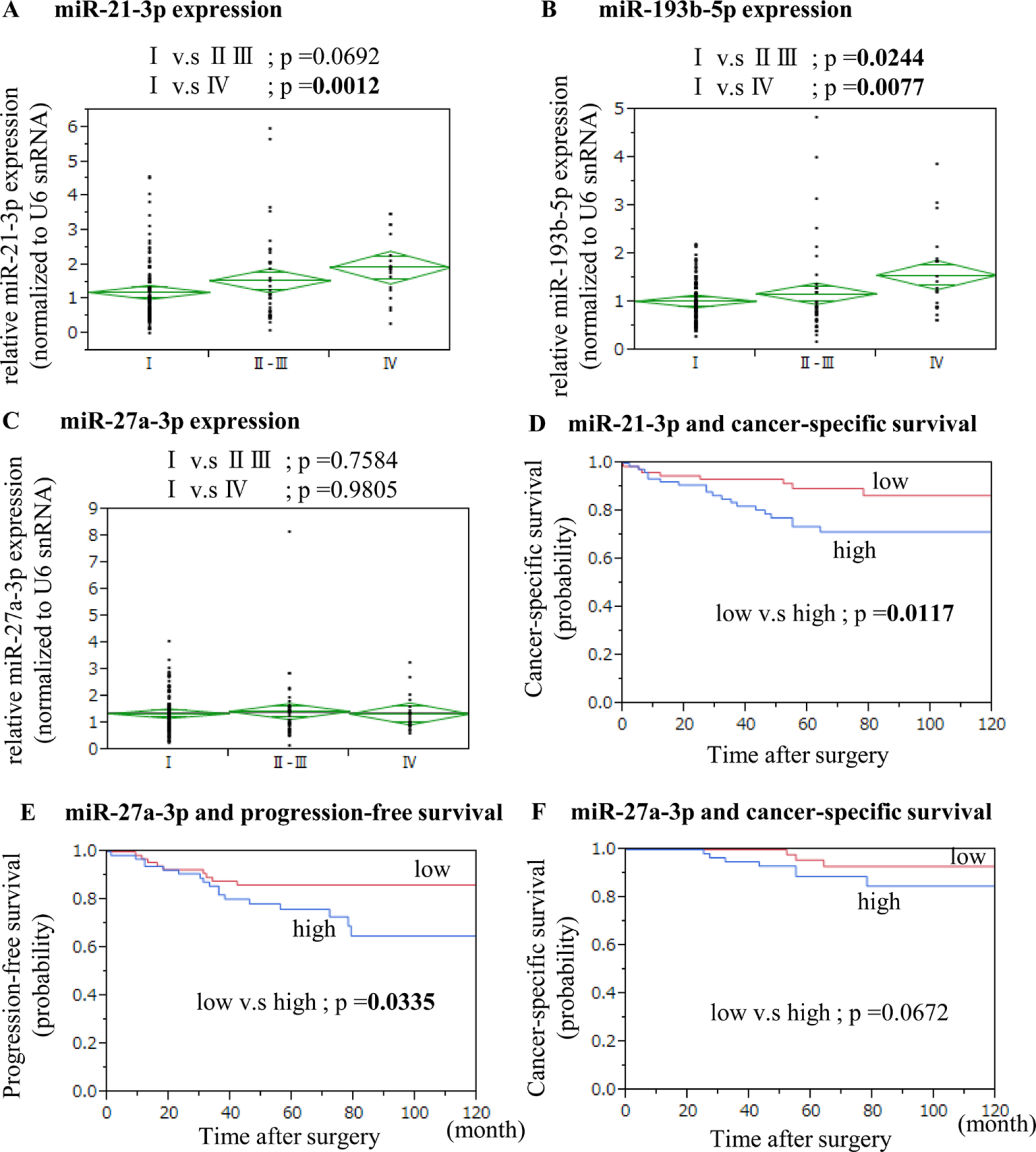
The association of miRNAs expression levels with TNM staging, cancer-specific survival and progression-free survival Levels of miR-21-3p **A.** and miR193b-5p **B.** differed significantly based on tumor staging (Wilcoxon test). However, there was no significant correlation associated with miR-27a-3p expression **C.** We divided the samples into two groups, high or low levels, based on real time PCR results. High expression of miR-21-3p **D.** was significantly associated with cancer-specific survival (Kaplan-Meier, log rank test). High levels of miR-27a-3p were positively associated with recurrence **E.** and cancer-specific survival **F.** in ccRCC (Kaplan-Meier log rank test).

The validation cohort comprised a total of 159 ccRCC samples obtained from patients with ccRCC between 2000 and 2009 (Table 1, Supplementary Figure 1). MiR-21a-3p, 193b-5p and 27a-3p were significantly up-regulated (*p* < 0.05), and miR-193a-3p was statistically up-regulated (*p* < 0.1) in the DOD/AWD group compared to the NED group (Supplementary Table 2). Furthermore, high levels of both miR-21-3p (Figure 1A) and 193b-5p (Figure 1B) were found to be associated with TNM stage in ccRCC. However, there were no significant correlations associated with miR-27a-3p (Figure 1C), 193a-3p and 34a-5p (data not shown) expression. Nuclear grade was not associated with the expression levels of any of these miRNAs (data not shown).

### MiRNA expression and cancer-specific survival in validation cohort

Using Kaplan-Meyer analysis and log-rank test, we found that levels of miR-21-3p (low vs. high *p* = 0.0117) showed significant association with cancer-specific survival rate (Figure 1D).

Univariate and multivariate Cox proportional hazards analysis were used to further evaluate the association of the 5 miRNAs with cancer-specific survival rate (Table 3). In univariate analysis, high levels of miR-21-3p (low vs. high HR, 2.75; 95%CI, 1.25–6.65; *p* = 0.0108), TNM stage (I vs. IV HR, 194; 95%CI, 50.4–1295; *p* < 0.0001, I vs. II, III HR, 12.19; 95%CI, 3.04–81.0; *p* = 0.0002) and histopathological nuclear grade (G1, 2 vs. G3, 4 HR, 6.12; 95%CI, 2.68–13.2; *p* < 0.0001) were significantly associated with cancer-specific survival rate. Venous invasion in the ccRCC specimens (positive vs. negative HR, 2.71; 95%CI, 0.95–6.78; *p* = 0.0593) was also correlated with patients’ prognosis, whereas, age, gender, and the levels of miR-193a-3p, 193b-5p, 34a-5p and 27a-3p did not demonstrate prognostic significance. Among the parameters shown to be significantly associated with cancer-specific survival rate by univariate analysis- TNM stage, histopathological nuclear grade and levels of miR-21-3p, only TNM stage (I vs. IV HR, 223; 95%CI, 49.3–1787; *p* < 0.0001, I vs. II, III HR, 12.3; 95%CI, 3.01–82.3; *p* = 0.0003) was significantly associated with patients’ prognosis when analyzed by multivariate analysis. Histopathological nuclear grade (G1, 2 vs. G3, 4 HR, 2.16; 95%CI, 0.91–4.92; *p* = 0.0794) tended to correlate with prognosis, whereas high levels of miR-21-3p did not have prognostic value.

**Table 3:** Univariate and multivariate Cox regression analysis on microRNA expression levels with cancer-specific survival in validation cohort (*n* = 159)

	Univariate		Multivariate	
Prognostic factor	HR (95% CI)	*P* value	HR (95% CI)	*P* value
**Age (≧65 versus < 65)**	1.29 (0.61–2.80)	0.4991		
**Gender (Female versus Male)**	1.38 (0.63–2.92)	0.4069		
**TNM Stage (II, III versus I)** **(IV versus I)**	12.2 (3.04–81.0) 194 (50.4–1295)	0.0002 < 0.0001	12.3 (3.01–82.3) 223 (49.3–1787)	0.0003 < 0.0001
**Grade (G3, 4 versus G1, 2)**	6.12 (2.68–13.2)	< 0.0001	2.16 (0.91–4.92)	0.0794
**Vein invasion (yes versus no)**	2.71 (0.95–6.78)	0.0593		
**miR-21-3p (high versus low)**	2.75 (1.25–6.65)	0.0108	0.58 (0.21–1.66)	0.3042
**miR-193a-3p (high versus low)**	0.91 (0.42–1.93)	0.8040		
**miR-34a-5p (high versus low)**	1.36 (0.42–2.94)	0.4162		
**miR-193b-5p (high versus low)**	1.87 (0.87–4.22)	0.1053		
**miR-27a-3p (high versus low)**	1.21 (0.57–2.60)	0.6215		

### MiRNA expression and progression-free survival in validation cohort

We next evaluated the levels of 5 miRNAs for M0 patients with ccRCC (*n* = 140) at the time of nephrectomy (Table 1). Using Kaplan-Meyer analysis and log-rank test, the levels of miR-27a-3p (low vs. high *p* = 0.0335) showed a significant association with progression-free survival rate (Figure 1E), while the levels of miR-21a-3p, 34a-5p, 193a-3p and 193b-5p did not. Furthermore, we showed that miR-27a-3p levels (low vs. high *p* = 0.0672) trend with cancer-specific survival in this cohort (Figure 1F).

Subsequently, a univariate Cox proportional hazard regression model was performed to determine the influence of miRNA expression, as well as of clinicopathological characteristics, on recurrence. TNM staging (I vs. II, III HR, 7.76; 95%CI, 3.54–18.27; *p* < 0.0001) and miR-27a-3p levels (low vs. high HR, 2.33; 95%CI, 1.07–5.47; *p* = 0.0330) showed significant association with cancer progression, and miR-193a-3p levels (low vs. high HR, 1.93; 95%CI, 0.90–4.37; *p* = 0.0942) showed a tendency to associate with cancer progression. Age, gender, histopathological nuclear grade, vascular invasion, and the levels of miR-21a-3p, 34a-5p and 193b-5p did not associate with cancer progression (Table 4). A multivariate Cox proportion hazard model, based on TNM staging and high levels of miR-27a-3p, revealed that miR-27a-3p expression (low vs. high HR, 2.71; 95%CI, 1.23–6.42; *p* = 0.0131) was a significant prognostic factor (Table 4). These results indicate that miR-27a-3p expression is a good independent prognostic biomarker to predict cancer recurrence.

**Table 4:** Univariate and multivariate Cox regression analysis on microRNA expression levels with progression-free survival in validation cohort (*n* = 140)

	Univariable		Multivaliate	
Prognostic factor	HR (95% CI)	*P* value	HR (95% CI)	*P* value
**Age (≧65 versus < 65)**	0.76 (0.35–1.62)	0.4791		
**Gender (Female versus Male)**	0.88 (0.36–1.94)	0.7560		
**TNM Stage (II, III versus I)**	7.76 (3.54–18.3)	< 0.0001	8.39 (3.81–19.9)	< 0.0001
**Grade (G3, 4 versus G1, 2)**	1.73 (0.41–4.98)	0.4050		
**Vein invasion (yes versus no)**	1.86 (0.53–5.00)	0.2932		
**miR-21-3p (high versus low)**	1.38 (0.65–3.01)	0.4061		
**miR-193a-3p (high versus low)**	1.93 (0.90–4.37)	0.0942		
**miR-34a-5p (high versus low)**	0.97 (0.45–2.08)	0.9382		
**miR-193b-5p (high versus low)**	1.30 (0.61–2.83)	0.4976		
**miR-27a-3p (high versus low)**	2.33 (1.07–5.47)	0.0330	2.71 (1.23–6.42)	0.0131

### Function analysis of miR-27a-3p

**Figure 2:**
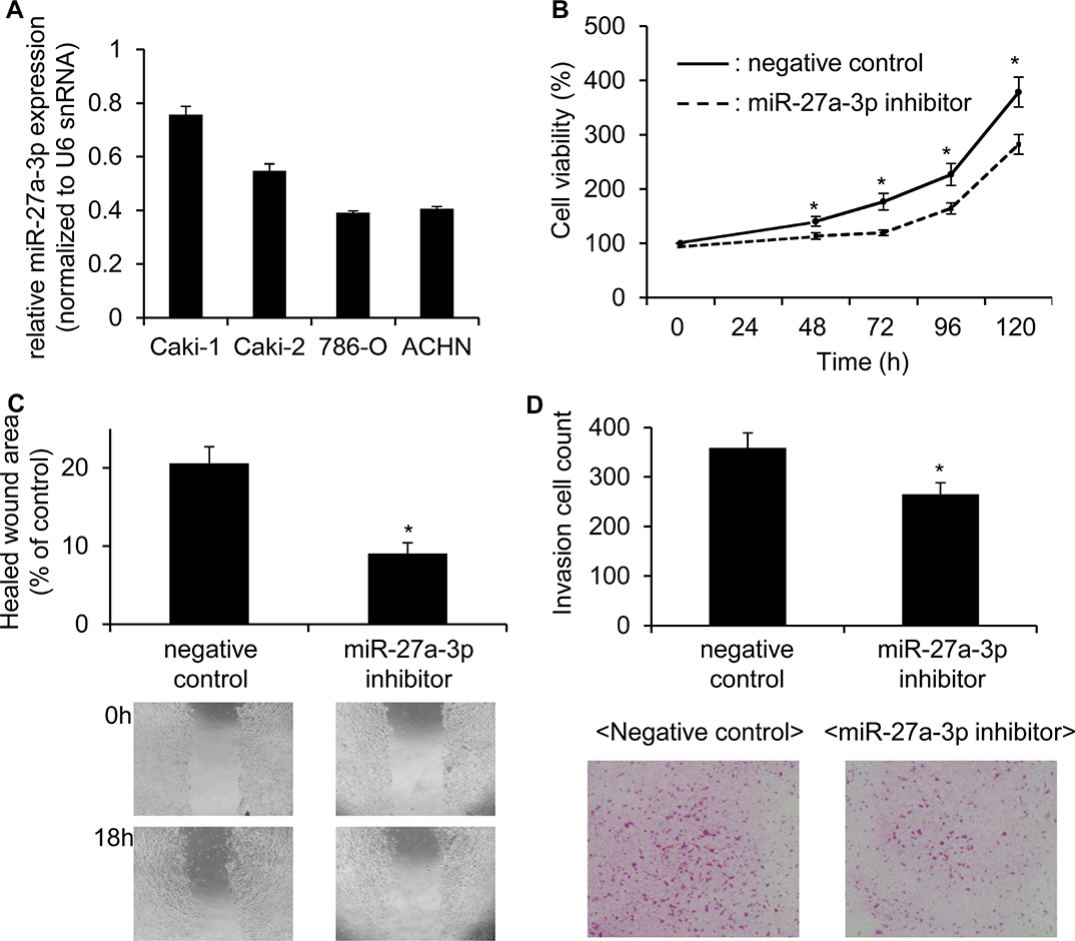
miR-27a-3p inhibitor significantly reduced the cell growth, migration and invasion ability in Caki 1 cells **A.** Expression of miR-27a-3p in ccRCC cell lines was examined by quantitative real-time PCR. **B.** The proliferation of Caki-1 cells transfected with the miR-27a-3p inhibitor or negative control miRNA inhibitor for 48, 72, 96 and 120 h were examined by MTS assay. Values are means ± S.D. of 6 independent experiments. *p < 0.05 vs. control inhibitor. **C.** Caki-1 cells were transfected with the miR-27a-3p inhibitor or negative control miRNA inhibitor for 72 h. Cell migration was measured 18 h after a wound was formed by scraping. Representative results of cell mobility in the scratch wound-healing assay are shown. The results are expressed as means ± S.D. of 6 independent experiments. *p < 0.05 vs. control inhibitor. **D.** Caki-1 cells were transfected with the miR-27a-3p inhibitor or negative control miRNA inhibitor for 72 h. The transfected cell suspension was added to the upper chamber of matrigel-coated transwell membrane inserts, and the lower chamber was filled with the complete medium. After 48 h incubation at 37°C, cells that had penetrated the matrigel were fixed with acetone and stained with H&E. Values are means ± S.D. of 6 independent experiments. *p < 0.05 vs. control inhibitor.

Next, we investigated the function of miR-27a-3p, since it may be a useful predictor for cancer recurrence. We first examined the expression levels of miR-27a-3p in ccRCC cell lines. In the 4 ccRCC cell lines (Caki-1, Caki-2, 786-O and ACHN) tested, we found that Caki-1 cells showed the highest miR-27a-3p expression (Figure 2A), and so we chose this line to study the effects of a miR-27a-3p inhibitor in terms of proliferation, migration and invasion.

MTT assays showed that inhibition of miR-27a-3p significantly reduced cell proliferation compared to a negative control (Figure 2B). Inhibition of miR-27a-3p also resulted in decreased cell migration by wound healing assay (Figure 2C) and decreased invasion as measured in an invasion chamber assay (Figure 2D).

**Figure 3:**
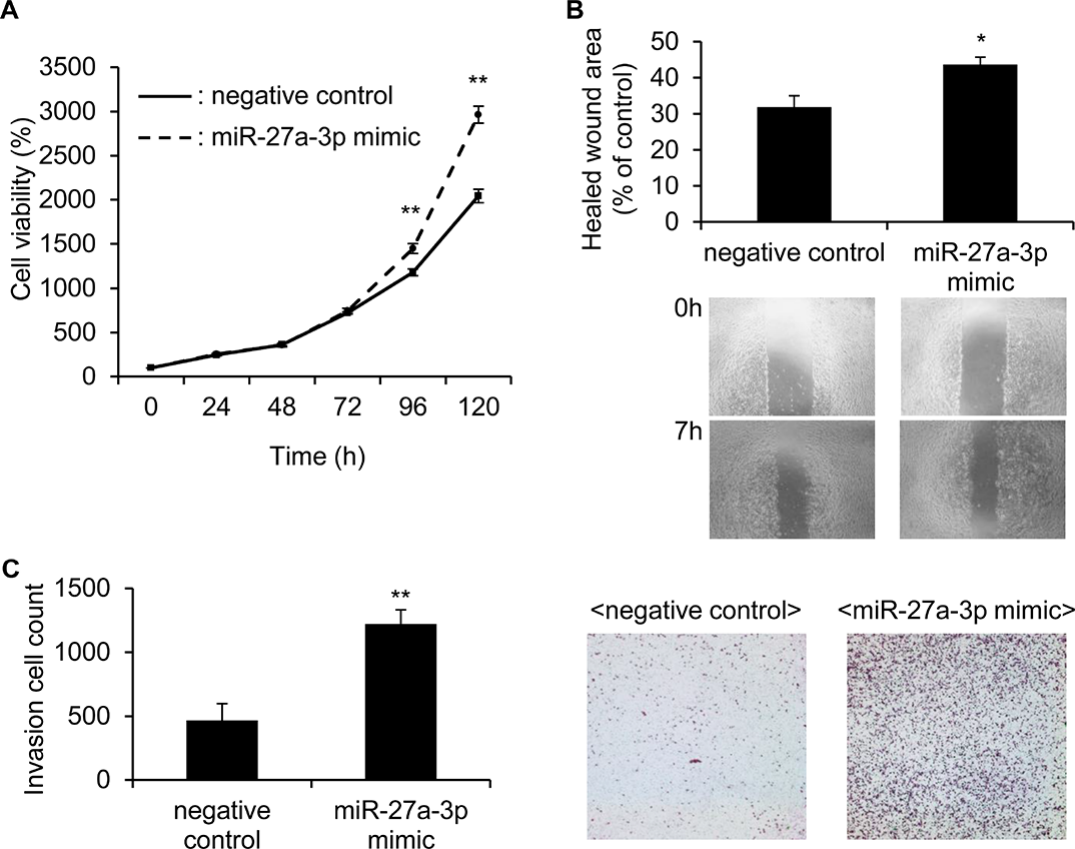
miR-27a-3p mimic significantly increased the cell growth, motility and invasion ability in 786-O cells **A.** The proliferation of 786-O cells transfected with the miR-27a-3p mimic or negative control miRNA mimic for 24, 48, 72, 96 and 120 h were examined by MTS assay. Values are means ± S.D. of 6 independent experiments. **p < 0.01 vs. control mimic. **B.** 786-O cells were transfected with the miR-27a-3p mimic or negative control miRNA mimic for 72 h. Cell migration was measured 7 h after a wound was formed by scraping. Representative results of cell mobility in the scratch wound-healing assay are shown. The results are expressed as means ± S.D. of 6 independent experiments. *p < 0.05 vs. control mimic. **C.** 786-O cells were transfected with the miR-27a-3p mimic or negative control miRNA mimic for 72 h. The transfected cell suspension was added to the upper chamber of matrigel-coated transwell membrane inserts, and the lower chamber was filled with the complete medium. After 18 h incubation at 37°C, cells that had penetrated the matrigel were fixed with acetone and stained with H&E. Values are means ± S.D. of 6 independent experiments. **p < 0.01 vs. control mimic.

Next, we examined the effect of a miR-27a-3p mimic on cell growth, motility and invasion in 786-O cells, which demonstrate the lowest miR-27a-3p expression among the 4 cell lines (Figure 2A). In contrast to the effects of the miR-27a-3p inhibitor, the miR-27a-3p mimic significantly increased the cell growth (*p* < 0.01; Figure 3A), motility (*p* < 0.05; Figure 3B) and invasion ability (*p* < 0.01; Figure 3C). These results suggest that miR-27a-3p could act as an oncomir in ccRCC cells.

## DISCUSSION

Metastatic ccRCC remains incurable, and all but one of the patients in our cohort with metastases at diagnosis died within 5 years after surgery. However, the prognosis for recurrent ccRCC varies widely. It has been reported that detecting early relapse can improve a patient's prognosis [7]. Thus, it is important to identify new biomarkers that, using surgical samples obtained during nephrectomy, predict cancer recurrence in ccRCC. Recent studies have demonstrated that miRNAs can play important roles in cancer development and progression for several types of cancer including ccRCC, and as such they may be useful as biomarkers and treatment targets.

In this study, we sought to identify oncogenic miRNAs in ccRCC by analyzing expression levels for 22 miRNAs in conjunction with patient prognosis in 159 ccRCC specimens. To our knowledge, there are only a few reports that focus on the association between specific miRNAs and cancer recurrence in ccRCC. Lower levels of miR-106b [13], miR-127-3p, miR-145, miR126 [14], and miR-30c [20] were significantly associated with low recurrence free survival rate. However, these studies included only small number of patients (*n* = 38–46) and lacked multivariate analysis. In this study, we demonstrate that miR-27a-3p is a novel useful independent biomarker in ccRCC to predict cancer recurrence.

It has been reported that miR-27a-3p is up-regulated in many cancers including ccRCC, and that high levels of miR-27a-3p lead to poor prognosis in breast cancer [22] and gastric cancer [23]. Osanto et al. [12] have reported that high levels of miR-27a in ccRCC were associated with poor overall survival; however this study included only 22 patients and lacked multivariate analysis. Another study, using 95 ccRCC samples, has shown that the expression of miR-27a-3p was significantly higher in nuclear grade 4 compared to grade 1 [21]. In our study, the expression of miR-27a-3p showed a tendency to associate with nuclear grade (G1–2 vs. G3–4; *p* = 0.06 data not shown). Furthermore, single nucleotide polymorphisms (SNP) of the miR-27a gene affect cancer risk in breast cancer [24] and renal cancer [16]. One SNP, rs895819, is located at the terminal loop of pre-miR-27a, and the variant allele of this SNP impaired the maturation of miR-27a and reduced its expression. Therefore, the variant allele of rs895819 resulted in risk reduction for breast cancer and renal cancer. These reports support the idea that miR-27a-3p might be a key molecule for ccRCC.

Two previous reports have performed functional analysis on miR-27a-3p in ccRCC. One report indicates that proliferation of SKRC7 cells was not affected by knock down experiments using a miR-27a-3p inhibitor [12]; however, another report claims that a miR-27a-3p inhibitor significantly suppressed proliferation in both 786-O and Caki-1 [25]. In our study, we demonstrated that inhibition of miR-27a-3p significantly reduced cell proliferation, migration and invasion ability in Caki-1 cells, and that a mimic of miR-27a-3p significantly increased cell proliferation, migration and invasion abilities in 786-O cells. Our data suggests that miR-27a-3p could act as an oncomir in ccRCC cells, and several studies of other human cancer cell lines supported these results. It has been shown that miR-27a regulates the signaling pathways of MET, EGFR [26] and MAP2K4 [27] and promotes cell proliferation, migration and invasion. Furthermore, it has been suggested that the expression of miR-27a was associated with the function of HIF-1α and HIF-2α, both of which are known to play an important role in renal tumorigenesis [28]. Together, these studies indicate that miR-27a-3p might function as an important oncomir in ccRCC and warrant further experiments to identify its molecular targets.

Although levels of miR-27a-3p were found to associate with progression-free survival rate in our study, they were not associated with cancer-specific survival rate. There are two possible reasons for this discordance, the first being related to molecular-target therapies. In Japan, molecular-target therapies, such as TKI and mTOR inhibitors, were introduced in 2009. Therefore, patients from both the pre- and post- molecular-target therapies era were included in this study. The second possibility for this discrepancy involves the presence of metastases, as levels of miR-27a-3p were not different between patients with or without metastasis at diagnosis. We did not find a strong connection between miR-27a-3p and cancer-specific survival rate, perhaps because miR-27a-3p is not predictive for presence of metastases.

In summary, we showed that high levels of miR-27a-3p were an independent prognostic factor for M0 patients in ccRCC. Therefore, it may be clinically relevant to assess miR-27a-3p expression in surgical samples at the time of nephrectomy to predict the risk of recurrence. Furthermore, miR-27a-3p could act as an oncomir in ccRCC cells and as such might be a candidate for targeted molecular therapy. However, because this study focused only on retrospective cases from a single institution, there is still a need to validate our results on other cohorts to definitively identify miR-27a-3p as an independent prognostic factor in ccRCC.

## MATERIALS AND METHODS

### Patient population

The study protocol was reviewed and approved by the appropriate institutional ethics committees. Tissue samples from patients who underwent nephrectomies at Osaka University Hospital between 2000 and 2012 and were histologically diagnosed as ccRCC were used in this study. None of the patients had received chemotherapy or radiotherapy before surgery. Histological diagnosis was determined on standard hematoxylin- and eosin-stained sections by 2 experienced senior pathologists. Patients were staged according to the 6th AJCC TNM staging system and tumors were graded according to Fuhrman's nuclear grading system. Survival data were available for all patients in the medical records at our hospital.

### Preparation of tissue samples and extraction of total RNA

These samples were obtained from nephrectomized ccRCC patients between 2000 and 2012. Samples obtained between 2000 and 2009 were frozen shortly after nephrectomy and stored at − 80°C; those obtained between 2010 and 2012 were immediately immersed in RNAlater (Qiagen, Valencia, CA, USA) after nephrectomy and stored at − 20°C. Total RNA was isolated using the miRNeasy Mini Kit (Qiagen) according to the manufacture's protocol. RNA integrity was confirmed by Experion (Bio-Rad, Hercules, CA, USA), and only non-degraded RNAs (RNA quality indicator > 7) were used in this study.

### Microarray analysis for miRNAs

MiRNA analysis was conducted using 9 ccRCC samples and matched-pair normal kidney samples on miRNA microarray 2.0 (Affymetrix, Santa Clara, CA, USA). The arrays were scanned using the Affymetrix Gene Chip Scanner 3000, and the scanned data were processed with the Agilent GeneSpring GX software (Agilent, Santa Clara, CA, USA) [18]. (http://www.ncbi.nlm.nih.gov/geo/query/acc.cgi?acc=GSE55138).

### Real time polymerase chain reactions (RT-PCR)

Expression of 22 miRNAs and small nuclear RNA U6 were measured using quantitative real time polymerase chain reactions (qRT-PCR). cDNA was synthesized using the mir-X miRNA first-strand synthesis kit (Clontech, Mountain View, CA, USA) according to the manufacturer's instructions. qRT-PCR was performed with a Thermal Cycler Dice Real Time System TP800 (TaKaRa Bio, Shiga, Japan) using SYBR premix Ex Taq II (TaKaRa Bio). Thermal cycling conditions included an initial step at 98°C for 30 s, and 40 cycles at 95°C for 2 s and at 63–66°C for 5 s for each miRNA-specific primer. We used U6 as a reference gene for normalization. PCR reactions for each sample were carried out in triplicate. After the reactions were completed, relative expression was calculated using the ΔC_t_ method, in which ΔC_t_ = C_t miRNA-X_ − C_t U6_.

### Cell culture

The Caki-1, Caki-2 and 786-O human RCC cell lines were purchased from the American Type Culture Collection (Manassas, Virginia, USA), and the ACHN cell line was kindly provided from Kyoto Prefectural University of Medicine. All cells were grown in RPMI supplemented with 10% fetal bovine serum (FBS) and 1% penicillin-streptomycin solution (Invitrogen Corporation, Oregon, USA), and incubated at 37°C in a humidified atmosphere containing 5% CO2. The medium was changed twice a week.

### MTS assay

Caki-1 cells or 786-O cells transfected with miRIDIAN hairpin inhibitor (Thermo Scientific Dharmacon, Buckinghamshire, England) or mimic (Thermo Scientific Dharmacon) were seeded in 96-well plates (Caki-1: 1.5 × 10^3^ cells/100 μL, 786-O: 1.5 × 10^2^ cells/100 μL) and allowed to adhere overnight. After 48, 72, 96 and 120 hours incubation, 20 μL of CellTiter 96 AQueous One Solution Reagent (Promega, Wisconsin, USA) was added to each well, the trays were incubated for 1.5 hours at 37°C, and the absorbance at 490 nm was measured with a microplate reader (Bio-Rad).

### Wound healing assay

Cell migration was evaluated using wound healing assays. In brief, Caki-1 cells or 786-O cells transfected with miRIDIAN hairpin inhibitor or mimic were seeded in a 24-well plate (2.0 × 10^4^ cells/well) and incubated for 72 hours. A wound was created in a monolayer of each line using a sterile 1-mL pipette tip at approximately 90% confluence, and cell migration pictures were recorded at 0 and 18 hours for Caki-1 cells and at 0 and 7 hours for 786-O cells.

### Invasion assay

The invasiveness of RCC cells was analyzed using a BD BioCoat Matrigel Invasion Chamber (pore size 8 μm) (BD, New Jersey, USA). Serum-free RPMI containing Caki-1 cells or 786-O cells transfected with the miRDIAN hairpin inhibitor or mimic (1.5 × 10^4^ cells) at 72 hours after transfection were introduced into the upper compartment in 500 ul medium; the lower compartment contained 750 μl RPMI with serum. After 48 hours for Caki-1 cells and 18 hours 786-O cells incubation at 37°C, cells that had penetrated the Matrigel were fixed with acetone, stained with H&E and counted.

### Statistical analysis

Statistical analyses were performed using JMP10 (SAS Institute Inc, North Carolina, USA). Results are presented as mean ± standard deviation (SD), and data were compared using the Wilcoxon test. Cancer-specific survival (CSS) and progression-free survival (PFS) were calculated using the Kaplan-Meier method, and differences between groups were assessed by log rank tests. Cox proportional hazards regression model analysis was performed to determine potential predictors of cancer-specific survival and recurrence. Differences were considered statistically significant when the *p* value was less than 0.05.

## SUPPLEMENTARY FIGURES AND TABLES


