# Supplementary material for: Expression of miR-27a-3p is an independent predictive factor for recurrence in clear cell renal cell carcinoma
Source: Oncotarget. 2015 May 27;6(25):21645–54. doi: 10.18632/oncotarget.4064 (PMC4673293; doi:10.18632/oncotarget.4064)
Supplement: Supplementary file 1 [file oncotarget-06-21645-s001.pdf]

SUPPLEMENTARY FIGURES AND TABLES

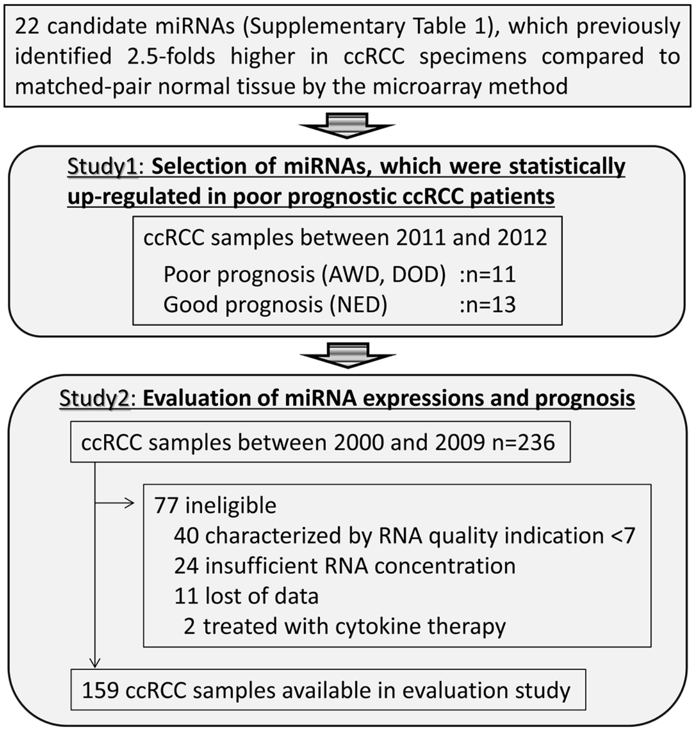

Supplementary Figure 1: Schema of the present study.

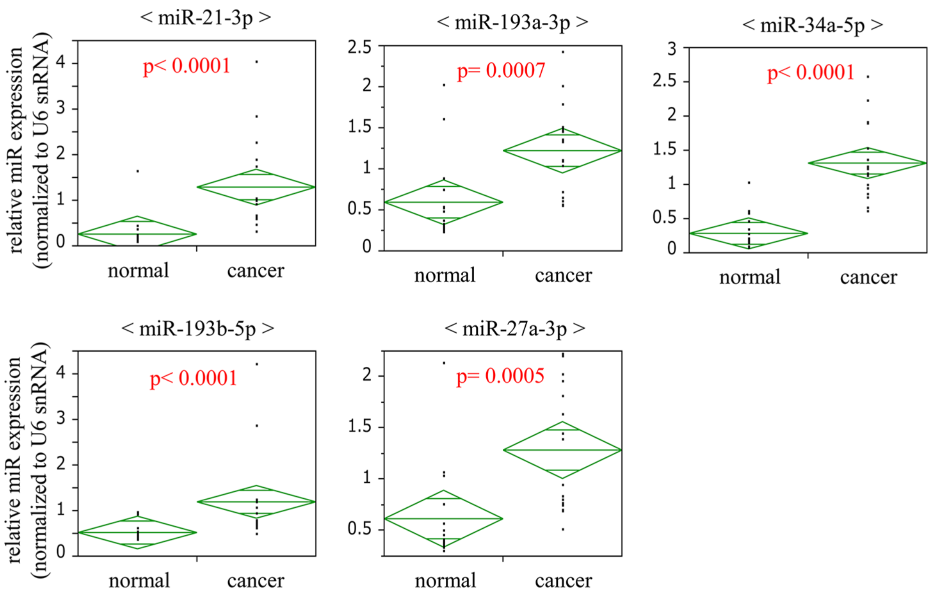

Supplementary Figure 2: Levels of 5 miRNAs were increased in ccRCC. A–E. The expression of each miRNA was examined in 16 matched-pair samples of ccRCC by quantitative real-time PCR. Statistical comparisons were made using the Wilcoxon test.

Supplementary Table 1: Microarray results showing miRNAs upregulated at least 2.5 fold in ccRCC.

miRNA	Chromosomal location	Fold change	*P* value
**miR-122–5p**	Chr18 q21.31	60.24	1.41E-06
**miR-34a–5p**	Chr1 p36.23	8.78	1.26E-07
**miR-210–3p**	Chr11 p15.5	8.22	1.34E-04
**miR-21–3p**	Chr17 q23.1	6.52	2.10E-05
**miR-155–5p**	Chr21 q21.3	6.29	4.77E-06
**miR-34a–3p**	Chr1 p36.23	6.02	2.18E-09
**miR-1271–5p**	Chr5 q35.2	5.01	3.12E-03
**miR-193a–3p**	Chr17 q11.2	4.68	1.31E-04
**miR-224–3p**	ChrX q28	4.31	4.22E-04
**miR-106b–3p**	Chr7 q22.1	3.73	1.40E-04
**miR-193b–5p**	Chr16 p13.12	3.69	6.80E-03
**miR-629–5p**	Chr15 q23	3.65	2.46E-03
**miR-92b–3p**	Chr1 q22	3.53	9.27E-07
**miR-3128**	Chr2 q31.2	3.52	4.98E-03
**miR-15b–5p**	Chr3 q26.1	3.32	4.64E-06
**miR-148a–3p**	Chr7 p15.2	3.25	5.62E-03
**miR-451a–3p**	Chr17 q11.2	3.11	3.87E-02
**miR-146b–5p**	Chr10 q24.32	2.74	6.68E-03
**miR-21–5p**	Chr17 q23.1	2.69	4.13E-02
**miR-27a–3p**	Chr19 p13.12	2.68	2.36E-04
**miR-15a–5p**	Chr13 q14.3	2.52	1.50E-06
**miR-148b–3p**	Chr12 q13.13	2.52	7.69E-03

Supplementary Table 2: Candidate miRNAs differentially expressed in validation cohort in two groups (NED vs AWD and DOD)

miRNA	NED Median (IQR)	AWD or DOD Median (IQR)	*P* value (Wilcoxon test)
**miR21–3p**	0.98 (0.60–1.61)	1.41 (0.70–2.35)	0.012
**miR193a–3p**	1.08 (0.56–1.50)	1.37 (0.71–1.86)	0.099
**miR34a–5p**	1.19 (0.82–1.76)	1.26 (0.87–2.03)	0.295
**miR193b–5p**	0.90 (0.63–1.27)	1.21 (0.74–1.83)	**0.015**
**miR27a–3p**	1.14 (0.73–1.58)	1.40 (0.97–1.85)	0.026

NED: no evidence of disease, AWD: alive with disease, DOD: dead of disease, IQR: interquartile range
